# An open invitation to productive conversations about feminism and the spectrum of eating disorders (part 1): basic principles of feminist approaches

**DOI:** 10.1186/s40337-022-00532-x

**Published:** 2022-04-19

**Authors:** Andrea LaMarre, Michael P. Levine, Su Holmes, Helen Malson

**Affiliations:** 1 School of Psychology, Massey University Auckland, Private Bag 102904, North Shore, Auckland, 0745 New Zealand; 2 Kenyon College, Gambier, USA; 3 University of East Anglia, Norwich, UK; 4 University of the West of England, Bristol, UK

## Abstract

Despite the long history of feminist research in the field and the clear relevance of questions of gender to this sphere, many continue to question the relevance of feminism for understanding and treating eating disorders in 2022. In this set of two papers, we explore some of the tensions, omissions and misconceptions which surround feminist approaches to eating disorders. At the core of these two papers is our assertion that such approaches can make significant contributions in the eating disorders field along six key lines: enriching the science of eating disorders, unpacking diagnostics, contextualizing treatment and prevention, attending to lived experiences, diversifying methodologies, and situating recoveries. In this first paper, we outline what feminist approaches are and dig into some key tensions that arise when feminist approaches come to the table. These include critiques of sociocultural approaches to understanding eating disorders, the relationship between feminist approaches and biological and genetic attributions for eating disorders, and the role of men. We then offer a key contribution that feminist approaches have made to eating disorders scholarship: an invitation to unpack diagnostic approaches and situate eating disorders within the landscape of food, weight, and shape concerns in the twenty-first century.

## Plain English Summary

Feminist research has been contributing to the eating disorders field for decades; yet, there continue to be questions about its relevance in 2022. In this set of two papers, we explore some of the questions around and disagreements about feminist approaches to eating disorders. We argue that feminist approaches to eating disorders continue to matter because they enrich the science of eating disorders, help us to better understand and situate diagnoses, consider treatment and prevention in context, attend to lived experiences, broaden our approaches to doing research, and consider recovery in context. In this first paper, we outline what feminist approaches are and dig into some key tensions around them. These include how sociocultural approaches to understanding eating disorders have been critiqued, relationships between “biological” and “feminist” understandings of eating disorders, and the role of men. We then consider one of the key contributions feminist approaches has made in the field: an invitation to think about eating disorder diagnoses in relation to contemporary concerns about food, weight, and shape.

## Introduction

Despite the increased visibility of feminism in scholarship and in everyday life, a reluctance to embrace feminism in psychology, psychiatry, and dietetic approaches to eating disorder research and treatment persists. A PsychInfo search for the period January 2019-October 2021 yielded only two articles [1, 2] with “feminism/feminist” and “eating disorder” (or one of the recognized eating disorders) in the title. Neither of those represented a feminist treatment or preventive intervention.

Within eating disorders journals in particular, the absence of explicitly feminist scholarship is certainly striking. The preeminent source in the field is the *International Journal on Eating Disorders,* first published in late 1981. A search of its 303 issues through to September of 2021 yielded only 3 articles with “feminism” or “feminist” in the title, and only 9 between 2011 and 2021 that have those terms anywhere in the article. Given that eating disorders have historically been perceived as ‘women’s issues’ and as primarily affecting women, this omission is quite remarkable. In addition, in their review of eating disorder models, Pennesi and Wade [3] found that no expressly feminist approach other than objectification theory met all four of their criteria for having received sufficient research attention and empirical support from various independent investigators. We speculate that objectification theory has become popular because, despite its feminist sociocultural foundation, it is more easily understood in *individualized* terms as a measurable, valid, and internal psychological attribute.

Nevertheless, there continue to be vibrant research and clinical communities working on feminist approaches to eating problems—particularly those grounded in intersectionality^1^ [4]—arguing that we *must* attend to structural and systemic factors that (a) constitute and reinforce sexist oppression; (b) impact experiences around food and exercise practices and embodied distress; and (c) affect the ways researchers, therapists, and activists think about what they are doing and why [5–7]. In this two-part series, we invite readers to join us in the process of answering two broad questions: (1) What issues and barriers have resulted in feminism being so neglected in the field of eating disorders? and (2) What can feminism offer?

In this article (part 1), our goal is to articulate the assumptions and principles that make an approach “feminist.” We do this by considering some definitions, reflecting on the history of feminism in the eating disorders field, and addressing some common misconceptions that are featured in critiques of feminism. We also illustrate some key principles, reflecting on how feminism approaches the important and always controversial matter of diagnosis. In the next paper, part 2, we explore in more detail what feminist approaches can offer the scientific endeavor to understand, treat, and prevent the spectrum of eating disorders. The second paper analyzes some of the tensions that we uncover in this first paper, namely how feminist approaches can invite a deeper engagement with social contexts in treatment and prevention. Hence we explore how feminist approaches enable us to (i) work with lived experience; (ii) expand on taken-for-granted but limited understandings of sociocultural influences; (iii) appreciate the value of engaging with different methodological approaches for understanding eating disorders; and (iv) illuminate the significance of context in understanding lived recoveries. This first paper sets the stage for this exploration, offering a discussion of and critical engagement with feminist literatures on eating disorders.

It is important to acknowledge up front two fundamental and interrelated feminist principles. First, everyone has a potentially important “voice”—that is, everyone has something potentially meaningful to express in relation to matters that affect them. Issues around who is allowed to speak, to have a legitimized voice, are important in research, clinical practice, and advocacy settings alike.^2^ Second, all too often important voices are excluded from conversations that directly affect them, and ultimately no one deserves or has “the final word.” Consequently, we do not expect that these two papers will constitute “the final words” on whether or why, as we believe, feminism belongs firmly entrenched in the eating disorders field. That said, we want to make our advocacy position clear: the neglect of feminism within mainstream eating disorder research and treatment is a serious omission that invites urgent consideration by scholars and clinicians alike. Therefore, we seek to de-mystify what we mean when we say that feminism *does* belong within the eating disorders field, and why we believe this. The heart of these two papers is our assertion that feminist approaches can make significant contributions in the eating disorders field along six key lines: enriching the science of eating disorders, unpacking diagnostics, contextualizing treatment and prevention, attending to lived experiences, diversifying methodologies, and situating recoveries.

## What are feminist approaches?

“Feminism” has often been taken as a singular force, and, like other terms adopted (often begrudgingly or even facetiously) into the popular lexicon, it suffers from a lack of specificity in use. Arguably, any singular definition of feminism does a disservice to the vitality and diversity of the movement [5, 8]. It may be more accurate to speak of feminism*s,* foregrounding a plurality of approaches. However, as Black feminist scholar bell hooks [8] noted, “without agreed-upon definition(s), we lack a sound foundation on which to construct theory or engage in overall meaningful praxis” (p. 18). Thus, we draw on hooks and others to provisionally define feminism here.

Broadly, as hooks articulated, “feminism is the struggle to end sexist oppression” and it “has the power to transform in a meaningful way all our lives” ([8], p. 26); rather than a lifestyle, it is a “political commitment” ([8], p. 28). From the perspective of research methodology, feminism combines a gendered lens with “a recognition of bias as an inherent aspect of any human inquiry, a stance of self-conscious reflexivity, and an emphasis on context as an essential factor in understanding behaviour” ([9], p. 439, citing Morawski, 1990). As explained briefly below, feminism is an overarching term for a set of different theories, and what feminism looks like in practice differs enormously based on contextual, practical, and sociohistorical factors.

Often, feminisms—particularly Western feminisms—are articulated as “waves.” This is seen as beginning with suffragette feminism (19th and early twentieth century, largely dominated by White women of at least moderate wealth and privilege), moving to “second wave” feminism (1960s through 1980s) aimed at explorations of the gendered dimensions of sexuality, work, family, and legal inequities, and toward “third wave” feminism (beginning in the early 1990s) with a focus on how intersecting aspects of identity, such as gender, ethnicity and social class, impact being-in-the-world. This “numbered wave” metaphor implies a linear path, but feminist praxis is not necessarily so neatly organized [10]. Indeed, this imposition of linearity may alienate groups of people engaged in feminist research and activism across generations [11, 12].

Moreover, within those “waves” there have been those who specifically endorse liberal (choice-based) feminism (e.g., [13]), relational cultural feminism (e.g., [14]), radical feminism (e.g., [15]), Marxist feminism (e.g., [16]), eco feminism (e.g., [17]) and more. These nuances matter when it comes to considering debates around the place of feminism in eating disorders work: rather than simply saying feminism does or does not “apply” as a framework, we ask: which feminisms, and to what end?

## Feminism and eating disorders: 1970—2000

Feminisms have been present within the eating disorders field for at least 45 years, that is, since the latter part of the second wave [18]. Feminist work has framed eating distress in relation to the social and cultural expectations surrounding female bodies, appetites, sexualities, social roles, and personal agency, highlighting how gender and other forms of inequality are culturally and politically important. Typically, this involves a critique of the idea of individual eating “pathology” as a personal deviation from cultural norms. Importantly, this does *not* mean denying the human impact and suffering associated with behaviors and thoughts that have come to be known around the same time, in DSM-III (1980), as the “eating disorders.”

Feminist accounts of eating disorders differ, including *within* the “waves.” Nevertheless, authors whose work followed on from Second Wave feminism largely attributed a rise in eating disorders to the pressures and contradictions relating to women’s roles, illuminated by the Women’s Movement. As with research on eating disorders even today, anorexia nervosa was prioritised here. Both Kim Chernin [19, 20] and Susie Orbach [21] focused on mother-daughter relationships as central (see also [22]); they identified how an “anorexic” may feel guilty about “abandoning” her^3^ mother (who was construed as repressed and unfulfilled); how she experienced abject terror around the potential of taking up a domestic, maternal role; and how she aimed to disassociate herself—bodily and socially—from the “female identity” sculpted by patriarchal society. These feminist analyses also considered desire—anorexia nervosa was seen, here, as a “solution” in a cultural setting wherein a woman felt “her own needs and desires intensely” (Orbach, [21]: xvii) despite being socialized not to. In this feminist model, anorexia nervosa was framed as a way for a woman to “negotiate conflicted desire” and fiercely assert control and autonomy by using body and food as metaphors (Orbach, [21]: xiv).

Although early feminist authors did not see eating problems as simply a product of media power or post-1960s concerns about “obesity”, they argued that a spectrum of eating distress logically results when culture foregrounds intensive surveillance over eating in the form of dieting as “normal” for women. Several feminist scholars (e.g., [23, 24]) considered the (increasingly thinner) thin ideal and its promotion to be a form of social control and political assault over and against women. Those experiencing eating issues, and anorexia nervosa in particular, were not only dis/figured as victims enmeshed in a patriarchal backlash: anorexia nervosa was positioned as an expression of contradictory cultural pressures [21, 25]. Situating eating issues within cultural contexts thick with gendered power differences, starvation was interpreted simultaneously as rendering femininity fragile, weak, and small *and* as a woman’s attempt to defiantly reject feminine subjectivity and escape into a childlike or de-feminised form [23].

As noted above, feminism’s historical and sociocultural perspectives have challenged the division between those who *have* or do *not* have eating disorders, instead positioning eating problems on a continuum with *normative* femininity [23, 24, 26, 27]. Crucially, this approach does *not* downplay the suffering that eating distress can engender. Rather, it challenges a strictly pathology-driven framework for evaluating origins, the seriousness of concerns, and the implications of the spectrum of distress for public health, social justice, and political change. Feminism situates eating disorders as “graphic cultural statement[s]” about “the ‘conditions of being a woman’ in contemporary western cultures... and... as expressive of a diverse range of sometimes contradictory societal values” that work to delineate the possibilities for women’s bodies and subjectivities ([28], p. 137). Although, as we will discuss, the sociocultural critiques from second wave feminists tended to privilege Western, White, heterosexual, and cisgender women, the importance of racism and classism in eating problems has been considered since the late twentieth century [29, 30], and more recent feminist work has explored marginalized sexual identities in eating disorder aetiology and experience [31].

Exploring eating distress in relation to gendered sociocultural contexts was, for many, not merely an academic pursuit or intellectual exercise. In most instances, feminist work in the field was developed from within clinical contexts by women who were practicing therapists or counsellors (e.g., [18, 32]; see also [33, 34]). As such, this was a theoretical and political project that aimed to *intervene* in how eating problems were understood, treated, *and* prevented. In this respect, there was certainly a good deal of early excitement, in research and treatment contexts, about the capacity for feminist approaches to extend our understandings of how to prevent and treat the distress many feel in their bodies and around food [23]. This was evidenced by theses, dissertations, articles, and book chapters (e.g., [35]) in the early 90 s proclaiming the revolutionary value of these approaches.

The zenith of this effort was the publication in 1994 of a 450-page (22 chapters) edited volume entitled *Feminist Perspectives on Eating Disorders* [33]. This enthusiasm was also the principal motive for the creation in 1990 of an emphatically feminist eating disorders conference, now in its 31st year, sponsored by the Renfrew Center Foundation in the USA. However, although work on feminist approaches to eating problems has expanded considerably since the earlier authors were writing – now representing a vibrant and active area of study [25] – they have not enjoyed the same scholarly *or* clinical uptake as approaches that engage more closely with individual psychopathology and diagnostic nosology and which focus more closely on individual cognitions and behaviours. Why?

## Addressing three major critiques of feminist approaches confining anorexia nervosa to a golden cage

Some of the resistance against, if not outright rejection of, feminist approaches stems from a broader critique of their emphasis on sociocultural factors in the development of eating disorders—including some of the points of theory we have noted above. Often, Hilde Bruch’s work (e.g., [22]) is taken as a starting point for approaches to understanding eating disorders that underscore the ways in which the sociocultural surround works its way into the psychopathology, including identity development, of a person whose transition into adolescence or young adulthood coincides with an eating disorder [36]. Somehow, Bruch’s work has come to be positioned as emblematic of feminist and sociocultural perspectives on eating disorders—despite it also informing, to a degree, some approaches that have become more “mainstream”, including the Maudsley model of family-based treatment and its successors [37].

Whether or not Bruch was a feminist therapist is an open question. On the one hand, Bruch’s stance could be characterized as feminist because she construed girls at risk as being imprisoned in a “golden cage” [22], and she coached therapists to adopt a “‘naïve’ stance that emphasizes listening to the patient and stimulating curiosity and sensitivity towards oneself” ([36], p. 179). On the other hand, Bruch’s work in the 1970s drew considerable professional and public attention to anorexia nervosa at a time when the biologically-oriented neo-Kraepelinian psychiatrists were coming to prominence in shaping the first appearance of an eating disorders section (including bulimia nervosa) in the 1980 publication of *DSM-III*. Thus, Bruch’s work, including her participation in the *DSM-III*’s Advisory Committee on Eating Disorders, was influential in legitimizing eating disorders as categorizable individual pathologies, a stance at-odds with much subsequent feminist theorizing, which tends to problematize the academic and clinical judgments that result in individualization of that which is deemed “abnormal.” Indeed, Saukko [38] suggests that, problematically, “Bruch was responsible for defining the anorexic as someone with an insufficiently autonomous self – a definition which is still alive and well in clinical practice, in self-understandings of anorexic women and in popular culture” (p. 64).

More troubling from a feminist lens, Bruch argued that mothers’ deficient parenting contributes to the development of anorexia in their daughters by “weakening of individual autonomy in relation to socio-cultural influences” ([39], p. 6). Family-based therapists are particularly critical of the fact that in Bruch’s work “families are viewed as unnecessary, or worse, as interfering” ([40], p. 275) with the patient’s need for healthy identity-development in the context of individual psychotherapy.

When assessing the relationship between Bruch’s influential writings and feminist approaches to eating disorders, it is more accurate to assert that aspects of her work were *taken up* by feminists interested in better understanding distress and dysfunction around food and in bodies. Till [39] notes that Orbach’s [41] innovative feminist work on anorexia shares Bruch’s emphasis on exploring how “social expectations of femininity” can contribute to tensions, including contradictory messages, experienced by girls and women about their bodies. This focus on sociocultural strictures, magnified in the intense microcosm of the nuclear family, that keep women small—and desiring smallness—has also come under fire for promoting an over-focus on media ideals, weight stigma, and body weight, shape, and image in prevention, treatment, and research. These features of some eating disorders have been construed as a dangerous distraction from the neuroscientific and biogenetic (i.e., the neo-Kraepelinian) approaches that position eating disorders as medical pathologies first and foremost and only secondarily, or not at all, as sociocultural artefacts [42]. However, as we elaborate in the next section, feminist work is not necessarily in conflict with these neuroscientific or biogenetic approaches.

## Eating disorders are “serious biological illnesses”

As we move into this section, we acknowledge that “feminist” and “biological” approaches to understanding and addressing eating disorders do not live at opposite ends of a spectrum. Biological approaches to eating disorder treatment, prevention, and research rarely *completely* neglect sociocultural factors. For instance, the very premise of epigenetic research on eating disorders rests on the interplay between genes and environment [43], which necessarily implicates the sociocultural. Similarly, dominant aetiological explanations and treatment models for both bulimia nervosa and binge eating disorder give some attention to cognitions that derive from sociocultural values surrounding body weight and shape [44]. As we will argue, these perspectives need not be binarized at all. Nevertheless, “what we know” about eating disorders has often been informed by a perspective in which sociocultural factors are viewed as secondary rather than as fundamental aspects of research, prevention, and treatment.

In contemporary articulations of eating disorders, particularly anorexia nervosa, feminist issues are often positioned as secondary to responding to the immediate nutritional needs of those with eating disorders—focusing on ‘re-feeding the brain’ such that the person is restored to a state of health [45]. Feminist perspectives are seen as neglecting the real suffering and severity of the real illness in favour of more abstract readings of gender politics as they play out in “individual” psychopathology [23]. A more general and popular framing that effaces feminist concerns has been to emphasize that eating disorders are “biologically based mental illness[es],” a phrase favoured by many researchers, patients, families, and – perhaps ironically, given feminism’s emphasis on the sociocultural contexts of power – funding agencies and U.S. insurance companies [42].

An over-narrow focus on “biology” has undergirded a reliance on “valid” diagnoses of individual pathology and on genetics, temperament, neurochemistry, brain pathways, and the like. Concomitantly, feminist scholarship on eating disorders has been framed as a mirror image of this; as being solely about cultural manifestations of misogyny, such as mass media or the sad history of male-dominated psychiatry’s crimes against women and against minorities. In this framing, feminist scholarship is seen as ignoring or minimizing “the science” of eating disorders and other psychopathologies, including “valid” (unbiased) diagnosis, genetics, pregnancy and birth complications, and other sources of individual vulnerability [6, 46].

There is, however, nothing inherently anti-neuro/science about systematically studying how social conditions (e.g., trauma, influences on attachment, weight stigma) may contribute to bodily distress (e.g., anxiety about control of one’s body) and stimulate development of maladaptive coping mechanisms (e.g., excessive dieting and exercising to manage anger, hunger, or sexual desire). Affirmation of the importance of science is not an insistence on one set of facts. It is a faith in the value of certain methodologies, including constant critical self-reflection about our methodologies, to produce information that converges on knowledge that is most helpful for the reciprocal relationships between people and societies. As we will elaborate in the second paper in this series, careful, self-reflective attention to methodologies and to the power and limits of theories is one of feminism’s core strengths. Thus, blending neuroscientific and contextually-oriented work, including feminisms, may help to generate rich and nuanced understandings of why and when some people develop distress around food and eating while others largely avoid this—producing understandings that either/or divisions will miss [47].

## Excluding and blaming men

One unsurprising criticism of feminist approaches involves the role of boys and men in theory and research. At the risk of oversimplification, this critical stance has, or at least implies, three unconnected or only-loosely connected themes. First is the contention that the field of eating disorders in general, and feminist researchers and theorists in particular, has overemphasized negative body image, disordered eating, and eating disorders in girls and women, to the exclusion of boys and men. Second is the contention that in situating female bodies, hunger, and eating issues in sociopolitical and historical contexts, feminists have pointed to the disciplinary structures of patriarchy and thus are essentially “blaming men” for the spectrum of issues – and that this is no better than blaming mothers for their daughters’ weakened autonomy and “anorexic” symptoms. Finally, there is the assertion that feminist eating disorder experts have been hypocritical in focusing on the construction of White women’s gender roles while ignoring the construction of not only men’s gender roles, but also trans and non-binary gender roles and experiences and the ways in which gender intersects with race, class, and other positions within the patriarchies.

While these are important concerns, they are also undeniably complicated. We feel all we can do here is provide some background in support of our proposition that**,** while feminism as a paradigm needs to keep improving, the leveraging of these concerns as “final words” on the end of feminisms’ role in eating disorders work represents oversimplifications that do not support improvement. Instead, we invite further dialogue about feminism’s place in the eating disorders field, and the way that feminisms can and should change to attend to the increasing recognition of the importance of intersectionality and gender diversity.

We first consider the last concern, which is arguably a key point around which feminist scholars—including ourselves—might be held to account. The question of “what about men with body image issues and/or eating disorders?” has been a core feature of critiques of feminist eating disorders work. In his series of textbooks on theories of personality, David Funder [48] offers a set of principles for working with paradigms such as psychoanalysis or social cognitive theory. One particularly useful “law” is that a paradigm**’**s greatest strength will simultaneously be its greatest weakness. Thus, in their extensive and in-depth investigations of the nature and multidimensional causes of eating disorders, body image, and disordered eating, feminist theorists and researchers—and the fields comprising eating disorders in general—have potentially overlooked these problems in men [49, 50].

One could argue that this oversight is ironic, if not egregious, because feminists have argued that gender identity and gender roles are constructed within the web(s) of sociocultural factors emphasized by feminist theories. Men’s gender identities and roles are constituted within those same networks, so feminist theories and methodologies are ideally positioned to shed light on the relationships between men’s body image issues, concerns about muscularity, fear of fat, disordered eating, and eating disorders. As we will explore below, it is also important for feminist work on eating disorders to extend beyond a gender binary view, incorporating close attention to the interweaving of gendered power with trans and non-binary experiences.

Historically, many feminists, though not all (see, e.g., [51–53]), have indeed overemphasized the thinness ideals and heteronormative “feminine” appearance concerns of young, White, able-bodied, middle-and-upper-class cisgender girls and women. In their insightful commentary on the how intersectionality must be considered in eating disorders research, Burke, Schaefer, Hazzard, and Rodgers [54] point out the Whiteness, femaleness, and socioeconomically privileged lens of much eating disorders scholarship. It is only relatively recently that research on trans and non-binary experiences of eating disorders has become more commonplace (e.g., [55]). This work is important, and overlooking gender diverse experiences of eating disorders has been a significant oversight in earlier feminist work. There is a high prevalence of eating disorders amongst transgender and non-binary people [56]. To move forward, there is a need to acknowledge the ways in which treatment based on a binary concept of gender can fail to meet the needs of trans people with eating disorders [57] who have reported the need for increased education amongst healthcare providers in relation to providing gender affirming care [58]. Collaborating with, and being led by, trans, non-binary, and intersex people to better understand approaches to research and treatment that honour experiences beyond gender and sex binaries should be a core concern for feminist scholars.

Likewise, feminisms are well suited to address the ways in which patriarchal gender norms may create significant stigma for men when they experience eating disorders, leading to many men suffering in silence [59] and to delays in referrals when they do seek help [60]. In offering a lens through which to view the entrenchment and circulation of power and gender norms in sociocultural settings, feminist approaches are well-suited to addressing how masculinity norms can contribute to the development, maintenance, and exacerbation of eating disorders amongst men [61]. Other areas of expansion for feminist scholarship include better understanding the intersections between age and gender; for instance, researchers have begun to explore eating disorders amongst older men in particular, a significantly under-studied phenomenon (e.g., [62]).

These shortcomings, which we explore further in our second paper, do not, however, erase several important facts. First, although the epidemiological findings^4^ vary and are complicated (compare, e.g., Cohn et al.’s [49] review to the findings of Zerwas et al. [63] and to the very recent review by van Eeden, van Hoeken, & Hoek, [64]), in many studies, particularly of adolescents and young adults, there are consistent and sometimes extraordinarily large women-to-men disparities in body dissatisfaction, disordered eating, and eating disorders. Moreover, there is a mountain of quantitative, qualitative, and meta-analytic research evidence indicating that a variety of lived experiences and psychosocial influences many women experience throughout the life course are directly related to the spectrum of eating issues (see, e.g., [6, 65, 66]). As Levine & Smolak note, “the body has different meanings because of gender-based differences in actual experiences interacting with the larger culture” ([67], p. 140). Importantly, gender does not operate in a silo apart from other spaces of sociocultural belonging—feminists have for many years noted that people with less social-economic-political power—including immigrants and ethnic minorities, people experiencing socioeconomic deprivation, LGBTQI+ people, disabled people, and women—face more questioning, monitoring, controlling, and exploitation of their bodies [68].

Distinguished therapist Dr. Amy Baker Dennis (personal communication, mid-October, 1986) has long maintained that “nobody is to blame for an eating disorder, *but* everybody has a responsibility to contribute to treatment and prevention.” We believe it is a gratuitous claim to criticize feminists for “blaming men for causing eating disorders.” However, if critics can pinpoint sources in the literature, in media, etc., that do substantiate this claim, then we feminists have an obligation to sustain a dialogue with that contention. And, conversely, we believe critics of feminism have a similar obligation to work with us to address the following.

There is no doubt that patriarchy—ranging from sexism in politics and business to the early sexualization and objectification of girls and young women—contributes to the “nervosa” of eating disorders, including negative body image, a sense of helplessness, dissociation from internal signals, self-objectification, and an intense, dysfunctional fear of fat (see, e.g., [65, 66]). This means that, as part of that system, women play a role in establishing and policing their own unhealthy practices—not in an intentional or deliberate way, but rather as a part of broader social systems of which we are all a part. *And* this fact should not obscure another equally salient one: Men and boys retain a dominant status in many cultural contexts that produce and sustain the spectrum of body dissatisfaction, disordered eating, and eating disorders. Thus, while men as a social category are not to blame for eating disorders, large numbers of individual men—fathers, brothers, friends, coaches, physicians, psychologists, dietitians, fashion designers, teachers, and so on—need to be held accountable for normative (e.g., fat shaming, sexual objectification) and non-normative (e.g., sexual assault) attitudes and behaviors that set the stage for a host of eating issues [69]. So too do people across the gender spectrum living within and circulating dominant discourses within surveillant societies.

## A key contribution of feminist work on eating disorders: unpacking diagnostics

Feminist scholars have long questioned both the scientific validity and the politics of diagnostic categories, encouraging a socially-situated reading of (dis)embodied distress, and calling into question the pathologizing language around eating disorders (e.g., [46, 25, 69]). This work encourages a close look at the range of people’s lived experiences in their bodies [23]. For most feminist eating disorder researchers, the science and politics of nosology, including labeling, should be continually questioned in order to better understand people’s experiences while preventing suffering and expanding the influence, that is, the voices, of people excluded from conversations and decisions very much affecting their own lives.

For most eating disorder researchers and clinicians, the person’s embodied experience of distress is paramount: lived experience and self-reported suffering “count” equally as much as, if not more than, a diagnostic label—a label that was always intended to be hypothetical and heuristic, serving as the opening decision in a long, complicated, and unstable process. Ideally, if people say they are miserable, isolated, and struggling, they are believed, respected, and taken seriously within their own frameworks and contexts. Once again, in this respect feminism takes people’s lived experiences of what are construed as “eating disorders” very seriously.

Focusing on people’s lived, embodied experiences within a patriarchal, sexualizing society does not mean either jettisoning a nosological system such as *DSM-5* or ignoring medical complications of eating disorders, the role of temperament in recovery, or genetic vulnerability to anxiety/depression. Rather, feminisms encourage close reading of the person’s experience, the therapist’s experience, *and* broader social forces that constrain who they (and people in general) feel they can be. And rather than prioritizing pathologizing discourses as “correct” and stable (e.g., “what are we going to do about the bulimia nervosa that Michael has?”), the emphasis is on understanding what distress feels like and leads to within a particular social context, and on what therapists and people seeking help can do together to generate, that is, embody, constructive changes.

In practice, feminist researchers and clinicians vary in their relationship to the diagnostic terminology around eating distress. Some reject the terms, adding a slash or parentheses to eating dis/orders or eating (dis)orders, in order to disrupt the primacy of diagnosis. Others use terms like eating distress, eating issues, or eating problems, all of which acknowledge a range or spectrum of dis/embodied experiences. Still others simultaneously retain and critique diagnostic labels in their writing. Retention is done for varied—and not always articulated—reasons, ranging from aiming to speak authoritatively within spaces where feminist work is not always heard (e.g., [70–72]), to acknowledging the utility of the power that labels hold in terms of facilitating access to treatment in the twenty-first century, to working from a feminist paradigm that is not rooted in deconstruction and thus is perceived as more pragmatic. Note that within a feminist approach the multidimensional relationships between being told what you “have” (e.g., bulimia nervosa), constructing your identity, confronting stigma, and developing hope become important issues for both recovery and research.

Interrogation of the power, scientific validity, and possible danger of diagnostic labels is not new, nor is it unique to the eating disorders field or to feminism. Arguably, the eating disorders field lags behind other areas of mental distress, where diagnostic labelling and its reification have been points of contention tied to the exercise of power and control for at least 60 years (e.g., [46, 73, 74]; see also [75, 76]). Diagnostic labels rely at least in part on some form of self-report and in that sense acknowledge the importance of lived experiences, but the labeling process (situated in professional, clinical, and public policy settings) also reflects the operation of status and power in society and thus in science, including psychiatry and psychology [68, 77].

The concerns with eating disorder diagnostics raised by feminist scholars typically involve two key issues: (1) Although labels have the potential to convey understanding and hope for change, they also have the power to reduce people *to their diagnoses,* to the detriment of a sense of self beyond that diagnosis (e.g., [75, 78, 79]); and (2) The very instruments used to facilitate diagnosis of eating disorders are rooted in particular (white, young, thin, cisgender, heterosexual) populations [80–82]. With regard to the former, feminist researchers have written about the problematics of diagnoses within clinical settings, where the diagnosis can come to eclipse the person’s subjectivity, dictating behaviour toward and understandings of the person *as their diagnosis* (e.g., [79, 83]). Feminists have proposed that there is more value in looking beyond the diagnosis, which is always a “working” hypothesis about what a person “has”, to understand each person as they experience themselves in their gendered sociohistorical and cultural contexts (e.g., [23, 26, 79, 84]).

Such explorations in clinical training and in research are important. They remind us of a fundamental point made by sociologists such as Goffman [65] well over 50 years ago: essentializing diagnostic framings (e.g., calling someone “the anorexic” or “the bulimic” – or even a “patient”) come laden with assumptions and stereotypes—and power of the framers to say (i.e., to voice) what comes next [85, 86]. In critical feminist (often post-structural) accounts of eating disorders in particular, there is an emphasis on the power of language. The act of referring to someone diagnostically has material impacts on what they might imagine about themselves, as well as what others imagine about them. This may be particularly true of eating disorders, which often emerge during periods critical for identity development. There is also a recursive power-loop in terms of seeking and obtaining diagnosis: in the same way that the diagnosis carries implications for how the subjectivities of those diagnosed will be interpreted, so too do the politics of care and diagnosis impact who has access to those categories in the first place, let alone how they will be used.

With regard to the second point, the very instruments, including *DSM-5*, used in diagnosis may reinforce particularized versions of whose suffering is validated and whose is not attended to. For instance, measures held as the “gold standard,” such as the EDE-Q, “are based on presumed cisgender men and women and have not intentionally included transgender people” ([82], p. 1). In a confirmatory factor analysis exploring gender and ethnicity, McEntee et al. [81] discuss how the original four-factor, 22-item solution for the EDE-Q may not reflect gender and ethnicity differences in scores. Others have noted that the EDE-Q leads to different norm scores across populations and even *within* different subcultures in a Western context [80].

Further, feminists (e.g., [29, 87]) have, for decades, demonstrated the ways in which the cultural schemas used to establish the realities of clinical diagnoses of eating disorders and their attendant “psychopathology” may not adequately account for experiences that differ across cultures and/or within cultures as a function of ethnicity, social class, sexual orientation, and other relevant social positions. From at least the late 1970s and the development of *DSM-III*, the instruments and conceptual schemas used to diagnose eating disorders were established using populations (i.e., thin, White, cisgender, heteronormative, young women) that became the stereotype of eating disorders. This stereotype in large part reflected who was initially present for treatment and investigation as the field was being constructed and thus struggling to be recognized as relevant and “real.” It is well established that those who do not fit this profile often face barriers to diagnosis and treatment, including financial and cultural barriers [88]. General practitioners and specialists (e.g., in internal medicine, gastrointestinal disorders, obstetrics/gynecology), as well as dietitians, also receive minimal training in mental health and eating disorders [89, 90], so they may miss the signs of eating disorders, especially in people who do not fit societal stereotypes. If disorder is “detected” and a label applied, people whose bodies do not fit the expected eating disorder (for instance, those who are not young, White, thin, and cisgender women) are often labelled “atypical” or as having “disordered eating” (e.g., [91]), perhaps further entrenching their sense of being abnormal (un/re/cognized) within a system that continues to privilege the young, White, thin, cisgender, able-bodied, and rich.

Even scientist-practitioners working within more dominant frames in the eating disorders field suggest that our labelling (nosological) strategies are inadequate and require frequent revisions [92, 93]. Yet, there is hesitancy to either abolish categorization entirely or to embrace an approach that values “ill” people’s lived experience as an equally valid form of expertise.^5^ There is — even more strongly —a reluctance to know and voice the complex history of science and re/cognize and study how existing systems continue to uphold broad social marginalization and power inequities (see, e.g., [77, 68]).

Resistance to this critical framework likely reflects, at least in part, a more general suspicion of a feminist paradigm that resists two fundamental assumptions of most people who have earned or seek the title “scientist.” First, feminists refuse to grant a priori privileges, including the power to determine who speaks or who is read, to “expert knowers” who search and advocate for singular truths (see, e.g., [94, 95]). Second, feminist critics see clinician—and researcher—fallibility and subjectivity, operating within systems that fertilize and nurture the very conditions that put people “at risk” for eating disorders, as more fundamental issues than just expected shortcomings due to inexperience, lack of skill, or human limits on information processing. As scientists and as philosophers, feminists continue to critique these attitudes and practices, which are grounded in over-simplified, misguided assumptions about “true” scientific knowledge as “rational,” value-free, unbiased, and “clear-cut” [96].

Feminist critique insists on continuous self-reflexive interrogation of one’s assumptions and practices, with an eye toward who is not being heard, what is not being acknowledged, and what builds layered understandings of differences while reducing exploitative, sometimes destructive disparities in power [94, 97]. The latter points us, for instance, to the ways in which psychiatry’s and clinical psychology’s embrace of the medical model’s fundamental assumptions and linguistic practices in relation to syndromes, diagnoses, comorbidity, course of illness, and treatment may disempower and disembody people whose eating disorders express and represent issues of helplessness, subjectivity, and loss.

This ongoing critique, framed by the other feminist assumptions that question current diagnostic practices in research and treatment, contributes significantly to experts’ resistance to feminist approaches as compelling ways of contextualizing and addressing embodied and disembodied distress. Attention to feminist concerns about the current science of eating disorders and what has been excluded from the existing research literature should foreground the field’s commitment to exploring the ways in which power and voice within various communities, including the scientific communities, impact eating disorder prevention, treatment, and research. Despite burgeoning research exploring the experiences of marginalized people with eating disorders (see, e.g., [54, 98]), many people continue to be left out of what experts (including the authors) “know to be true” about eating disorders—and to be left out of constructing the research that shapes this knowledge.

In saying this is an issue of power and privilege, we are not interested in blaming individual clinicians, researchers, or leaders (e.g., of *DSM* work groups and other professional organizations) for neglecting to include people in larger bodies, racialized people, disabled people, LGBTQI + people, and others in their research and/or treatment approaches. Instead, we see these exclusions as emblematic of power systems that exclude some from being seen and heard, in clinicians’ offices, in the journals, in pools of potential research volunteers, and in the conferences, graduate training programs, and other sites in which legitimacy, including legitimacy (authority) in science, is constructed. These are systems of authority in which we are all implicated, and we need to examine how they play out in mental health prevention, treatment, and research in general and the eating disorders field in particular.

## Summary and conclusions

For decades feminist approaches to the spectrum of eating disorders have been an unwelcome presence in these fields, with the notable exception of objectification theory’s significant impact on our understanding of the effects of sociocultural factors such as media and peer influences. This explicit and implicit exclusion has unfolded in the context of various topics emphasized by feminism (e.g., media effects, gender differences, the importance of lived experience, sexual assault) becoming mainstream areas of study in the field of eating disorders. We have argued that this exclusion, which is based on a variety of misconceptions and historical sociopolitical factors, is not only unwarranted, it is detrimental to the science, practice, and advocacy that are at the heart of the eating disorders fields. Like all fields, the eating disorders fields need to address an expanding array of disorders and of peoples whose sufferings need to be acknowledged, while somehow identifying the shortcomings and lacunae in the approaches that have constructed and legitimized the field. We believe feminist approaches, while not without their own challenges, offer the capacities for self-critical analyses, improved science, and more productive advocacy—and thus deserve to be part of new, more respectful conversations.

## Data Availability

Not applicable.

## Abbreviations

DSM: Diagnostic and statistical manual
EDE-Q: Eating disorders examination questionnaire

## Footnotes

1 Intersectionality is praxis entailing the consideration of how various facets of social location commingle to create experiences of the world. Proposed by Kimberlé Crenshaw [4], this approach means acknowledging the nuances of human experience as they build and together create conditions of privilege and marginalization.

2 With this in mind, it is particularly important for us to also acknowledge our own positionalities as we write this piece. Particularly important is the acknowledgment that we are all White and from Global North, English-dominant countries. Three of us are cisgender women, and one of us is a cisgender man. We are at various career stages ranging from lecturer to professor emeritus, and we come from research, prevention, and advocacy contexts and do not practice clinically. Some of us identify as having lived experience of eating disorders or distress.

3 The individual with an eating disorder was typically assumed to be a woman at this time.

4 We acknowledge also the binary nature of much of this exploration and note that ongoing and future epidemiological research could be configured to engage more fully with experiences of gender and sex that extend beyond binary ideas about “men” and “women” or “males” and “females”.

5 It is worth noting that the RDoC criteria are increasingly gaining traction as a suitable way to understand and diagnose mental disorders including eating disorders. This domain (rather than symptom) based categorizing instrument provides more flexibility and possibly more individualization in categorization. However, it continues to rely on the heuristic of a spectrum from normal to abnormal and to classify individuals based on their deviations from norms, an approach questionable from the standpoint of understanding humanity itself as a collection of abnormalities.
